# Treatment efficacy of repositioning maneuvers in multiple canal benign paroxysmal positional vertigo: a systematic review and meta-analysis

**DOI:** 10.3389/fneur.2023.1288150

**Published:** 2023-11-06

**Authors:** Mohamad Alfarghal, Niraj Kumar Singh, Mohammed Abdullah Algarni, Nirmala Jagadish, Rajesh Kumar Raveendran

**Affiliations:** ^1^Otolaryngology-Head and Neck Section, Surgery Department, King Abdulaziz Medical City, Jeddah, Saudi Arabia; ^2^All India Institute of Speech and Hearing (AIISH), Mysore, India; ^3^King Saud Bin Abdulaziz University for Health Sciences, Jeddah, Saudi Arabia; ^4^King Abdullah International Medical Research Center, Jeddah, Saudi Arabia

**Keywords:** treatment efficacy, repositioning maneuvers, BPPV, multiple canal involvement, bilateral BPPV

## Abstract

**Introduction:**

Benign paroxysmal positional vertigo (BPPV) involving the posterior canal is more common than other canals; however, simultaneous involvement of multiple canals can be seen up to 20% of all BPPV cases. The diagnosis and management of multiple canal BPPV can be quite challenging due to the complexity of findings. Therefore, this systematic review and meta-analysis aimed at unveiling the most effective repositioning strategy for the treatment of multiple canal BPPV.

**Methods:**

A literature search through PubMed, Scopus, and Web of Science databases was conducted using search terms such as BPPV, multiple canals, bilateral BPPV, repositioning maneuvers etc. After duplicate removal, the retained articles underwent various stages of elimination by two independent reviewers, and a third reviewer resolved the discrepancy between them.

**Results:**

A total of 22 articles were included in the systematic review. These publications documented 5,196 patients diagnosed with BPPV, of which 513 had multiple canal BPPV. Of 295 individuals with multiple canal BPPV, 58.9% were effectively treated in 1 session, whereas 18.3 and 4.4% achieved a symptom-free state after two and three sessions, respectively. Failure of treatment using repositioning maneuvers was found in 18.4%.

**Possible implications:**

This study offers insight into the real world of BPPV management in single and multiple canal BPPV. It is evident that repositioning maneuvers provide rapid and long-lasting relief of BPPV in most single canal BPPV patients; however, multiple canal BPPV often requires repeated treatment, and the risk of recurrence is higher in this variety than the single canal BPPV.

## Introduction

Benign paroxysmal positional vertigo (BPPV), a disorder of the inner ear, is characterized by brief episodes of mild to intense vertigo triggered by specific changes in head position in vertical or horizontal planes (1, 2). BPPV ranks highly among the most common disorders of the vestibular system, accounting for nearly one-third of the vestibular disorders. The incidence of BPPV usually ranges from 10.7 to 140 per 100,000 people per year, depending on the population (3). It reaches a lifetime prevalence of 2.4% and shows a 1-year prevalence of 1.6% (4).

Benign paroxysmal positional vertigo has been explained by the theories of “cupulolithiasis” and “canalithiasis” (5–9). According to the canalolithiasis theory, the free-floating otoconia in the semicircular canal (SCC) causes exaggerated fluid movements toward or away from the cupula, leading to a transient stimulation of the sensory epithelia. As per the cupulolithiasis theory, the otoliths adherent to the cupula cause enhanced deflection of the sensory epithelium during head movements, leading to the perception of vertigo. Both theories are well accepted presently as they provide pathophysiological explanations for the different variants of BPPV.

The posterior SCC is the most common pathology site due to its greater reliance on gravity than other canals in both supine and upright positions (7, 10). Although the lateral canal variant of BPPV (LC-BPPV) is less common than the posterior canal BPPV (PC-BPPV), recent studies show a higher prevalence of LC-BPPV than the studies reported a few years or decades ago on this variant (11, 12). The anterior canal BPPV (AC-BPPV), multiple canal BPPV (MC-BPPV), and bilateral multiple canal BPPV (B-BPPV) are relatively rare variants.

The diagnosis of BPPV is based on the aggravation of vertigo associated with nystagmus on positional tests such as the Dix-Hallpike test and the Supine roll test (also called McClure-Pagnini test) (13). The presence of up-beating torsional nystagmus on the Dix-Hallpike test most often indicates a PC-BPPV, whereas down-beating torsional nystagmus tends to suggest an AC-BPPV. The presence of geotropic or apogeotropic horizontal nystagmus on a supine roll test calls for a diagnosis of LC-BPPV (14–16). The therapeutic management for BPPV includes Epley’s maneuver, Semont’s liberatory maneuver, Gufoni’s maneuver, Lempert’s maneuver, and Yacovino’s maneuver. The other less commonly used treatment maneuvers are Gan’s maneuver (17), modified Epley’s maneuver (18, 19), reverse Epley’s maneuver (20), and 360° somersault Epley’s maneuver (21) for PC-BPPV. For LC-BPPV maneuvers like forced prolonged procedure, Head shaking maneuver (22, 23), modified Gufoni’s maneuver (24), Cupulolith repositioning maneuver (25), Zuma maneuver (26), and modified Zuma maneuver (27) are also used. While there are several other options, the above-mentioned maneuvers are the most used options due to their efficacy (9, 28–34). All maneuvers rely either solely on gravity or inertia and gravity for removal of the otolithic debris from the semicircular canal and repositioning them in the utricle. The choices vary depending on the canal involved and also on the area in the SCC where the otolith debris is lodged (35). Sometimes, the choice is also dictated by convenience and personal preferences.

All the above-mentioned maneuvers have been found to work effectively in cases where the canal involvement is single. However, it is possible that two or more canals simultaneously have otolith debris. This situation is called MC-BPPV. A BPPV that simultaneously involves multiple canals is rare and usually affects canals in the same labyrinth (36). When bilateral canals are involved, it is termed B-BPPV. Multiple canal involvement has been observed in 6.8–20%, whereas B-BPPV accounts for 6–26% of individuals diagnosed with BPPV (20, 37–40). Tomaz et al. (39) reported that simultaneous involvement of the posterior and lateral canals was much more common than the involvement of the anterior and posterior canals or the anterior and lateral canals. However, for all practical purposes, the involvement of two or more canals on the same side, the same canals on both sides, or different canals on either side simultaneously can be subsumed under the umbrella of MC-BPPV (14, 20). The main etiological factors in cases with MC-BPPV are trauma and labyrinthitis (14, 39, 41).

Multiple canal BPPV is associated with severe clinical symptoms like persistent dizziness, balance issues, intense nausea and vomiting, and frequent falls (42). Multiple canal involvement often goes unidentified and underdiagnosed as it may exhibit various complex nystagmus patterns which might be confused for positional vertigo with a central source (41). The first-line treatment option for MC-BPPV is a repositioning maneuver suitable for the treatment of the semicircular canal that exhibits more severe symptoms and causes more intense nystagmus (43, 44). Unlike single canal BPPV, individuals with MC-BPPV were found resistant to treatment with standard canalith repositioning procedures (39, 45, 46). For example, in cases of B-BPPV, performing Epley’s maneuver simultaneously on both sides could end up being counterproductive due to higher chances of re-entry of the repositioned otolith particles back into the canal that was already treated or into another canal on the same side. The bilateral involvement influences the number of treatment sessions required to treat the symptoms due to which it is considered to have a less favorable prognosis than unilateral BPPV (47–50). However, if we look at multiple canals on the same side, individual canals exhibiting symptoms can be treated in a single session with a distinct time gap. Therefore, conceptual and procedural knowledge plays an important role in planning the most effective treatment.

Studies on single-canal BPPV have shown that the canalith repositioning procedure (CRP) remains an efficient and long-lasting non-invasive treatment of BPPV (51). The efficacy of some of the maneuvers mentioned above is well-established for single canal BPPV. However, the selection of an appropriate set of repositioning maneuvers for the management of MC-BPPV seems to be complex. The last decade has witnessed the use of several maneuvers for the management of individuals with BPPV; however, uncertainty persists regarding the combination of maneuvers to be used for the most effective treatment of MC-BPPV. Furthermore, there is no single study providing enough ground to understand the efficacy of maneuvers in treating individuals with MC-BPPV and recommend the best possible combination in comparison to the other. The above discussion points out clear gaps in the knowledge since there is no systematic review of the treatment efficacy of MC-BPPV in the concurrent literature. Therefore, the present systematic review aims to unveil the most effective combination of repositioning strategies in treating MC-BPPV.

## Methods

### Data sources and searches

The authors of the present systematic review performed a meticulous search across electronic databases, which included PubMed, Scopus, and Science Direct, to identify the studies on the treatment efficacy of repositioning maneuvers in MC-BPPV. The population, intervention, comparison, and outcomes (PICO) format was used to develop the search strategy. PICO is a format for developing a clinical research question by encompassing the four elements of a good clinical foreground question before starting one’s research.

The search terms used were: “*BPPV,*” “*multiple canal BPPV,*” “*repositioning maneuver,*” *and* “*treatment efficacy*.” The Boolean operators “*AND,*” “*OR,*” and “*NOT*” were used to create multiple search strategies. In Scopus and Science Direct databases, the search was done using the same search string. However, in the PubMed database, because of more specific indexing, the search was performed using the medical sub-headings combined with the Boolean operator “OR” in statements including “BPPV,” “multiple canal,” and “semicircular canal.” An example of the search string used in the PubMed database is (“benign paroxysmal positional vertigo”[MeSH Terms]) AND (“semicircular canals”[MeSH Terms] OR “semicircular canals”[Text Word] OR “semicircular canals”[Title/Abstract] OR “multiple canal”[Text Word] OR “multiple canal”[Title/Abstract]) AND (“repositioning”[Text Word] OR “repositioning”[Title/Abstract] OR “maneuvers”[Text Word] OR “maneuvers”[Title/Abstract] OR “treatment”[Text Word] OR “treatment”[Title/Abstract]).

The keywords and search strings were put together to create several permutations and combinations. Based on the relevance of the search results, a list of articles was obtained from each database. In addition, we imposed no constraints for the patient’s age or the time of the publication, except the last date on which the search was performed which was May 5, 2023. The resulting articles from each database were downloaded and saved in CSV or RIS formats. Later, they were uploaded to the Rayyan software (52).

### Study selection

Published studies that reported on the effectiveness of repositioning maneuvers in the treatment of MC-BPPV were eligible for inclusion. The inclusion criteria included studies using a positional test-based clinical diagnosis of MC-BPPV; retrospective or prospective case–control studies; studies that considered repositioning techniques as a possible management strategy; studies that evaluated the effectiveness of CRP, and studies with a well-documented number of treatment sessions for each patient and follow-up patients for recurrence. Exclusion criteria were, studies using non-human subjects, systematic reviews, meta-analyses, letters to the editor, case reports, or scientific conference reports; insufficient data on treatment or unclear diagnosis of MC-BPPV; studies published exclusively using a non-English language; studies with only a single canal BPPV, and studies with no availability/access to a full-length article.

### Procedure

**Figure 1:**
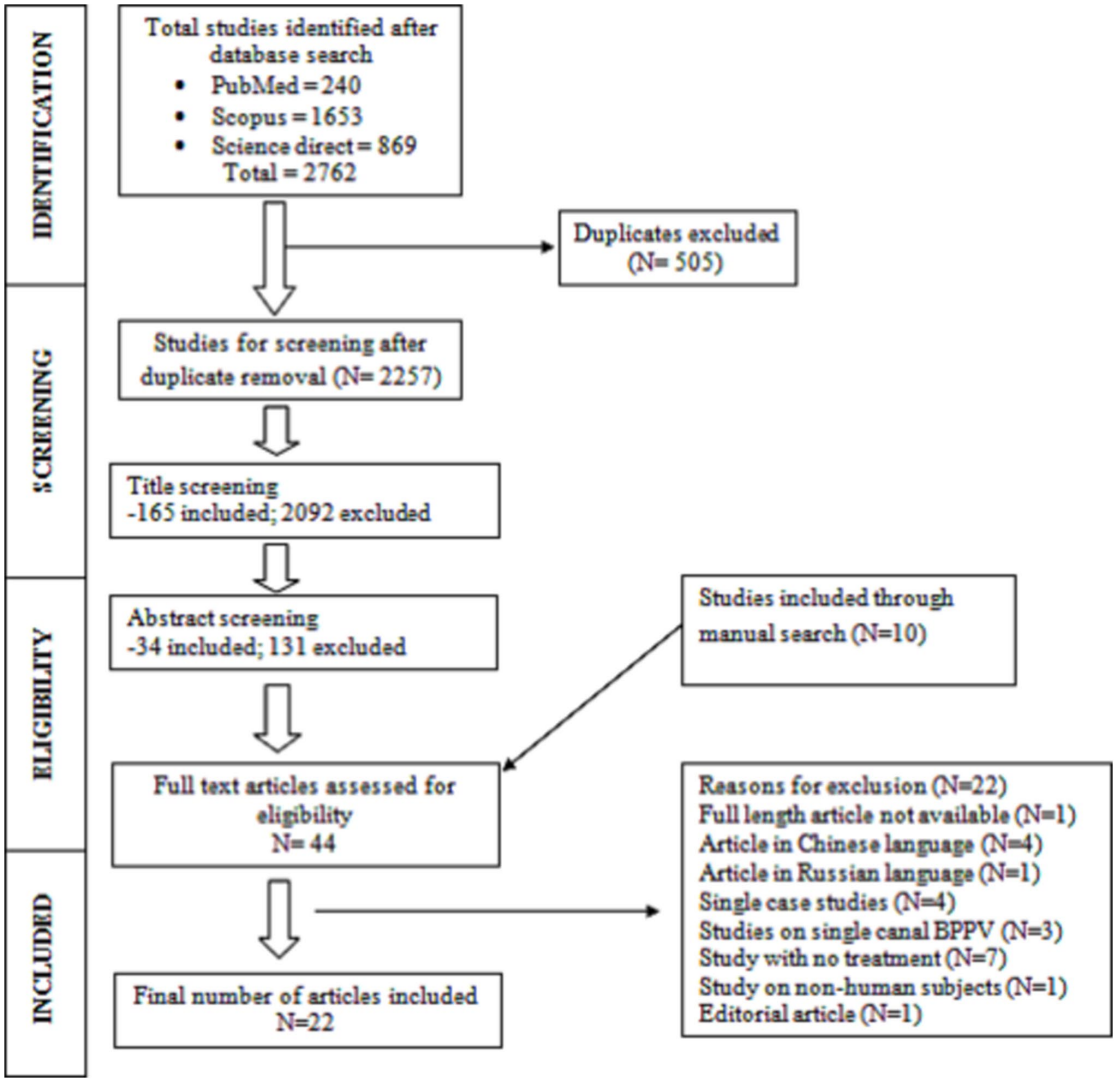
Flowchart of the study selection process adapted from the PRISMA flowchart (53).

Based on the inclusion and exclusion criteria, the research articles were included/excluded by the two reviewers (R4 & R5) using the Rayyan software. This software uses a blinded approach and hence each reviewer was unaware of the other reviewer’s decision. The title and abstract screening were carried out for all the articles. Of the 2,762 articles from the preliminary search, 1,653 were obtained from Scopus, 869 from Science Direct, and 240 from the PubMed database. After removing the duplicates (*N* = 505), 2,257 articles were retained as single copies. The title screening was carried out by R4 and R5 and the conflict in their decision was resolved through discussion. The conflict regarding the acceptability of a particular study was resolved by reviewer 3 (R3) in case the discussion was inconclusive between R4 and R5. After the resolution of the conflict by R3, a total of 165 articles were shortlisted in the title screening stage. These 165 articles further underwent abstract screening by R4 and R5. The conflict in their decision was resolved using the same strategy as in the title screening stage, which resulted in the retention of 34 articles for full-length screening eligibility. Also, the back-references of all 34 articles were referred to manually (snowballing) and 10 studies were found which was not retrieved in any of the search engines. Thus 10 manually searched articles were added to the full-length reading stage. Finally, 22 research articles were found to be suitable for this systematic review after a full-length review of all 44 articles. Twenty-two articles were excluded for various reasons mentioned in the Preferred Reporting Items for Systematic Reviews and Meta-Analyses (PRISMA) chart depicted in Figure 1.

### Data extraction from the selected articles

For each article meeting the inclusion criteria, information such as study setting, study design, patient selection process, treatment, outcomes, and results were extracted. The details regarding the article title, author, year of publication, type of research, and number of participants in MC-BPPV and single canal BPPV, the repositioning maneuver employed, number of treatment sessions provided, the follow-up duration, recurrence of BPPV, and canal conversion during treatment were also extracted from each article.

### Quality appraisal

Reviewers R1 and R2 performed each study’s quality assessment using the Newcastle-Ottawa scale (NOS). The NOS scale is an assessment tool to assess the quality of non-randomized studies, recommended by the Cochrane Collaboration. Patient selection, comparability, and outcome make up its three main elements. Each study is given a number between 0 and 9, and those with a score of at least six are acknowledged to be of high quality (54).

## Results

### Quality assessment

The average rating ranged from 6 to 8 on the NOS scale for all studies included in the present systematic review where higher values represent higher quality. The individual scores of NOS for each study are depicted in Table 1.

**Table 1:** Rating of each study on the Newcastle-Ottawa quality assessment scale for non-randomized studies in the systematic review.

Author(s) of the study and Year of publication	Selection				Comparability	Outcome		Quality
	Representativeness of the sample	Sample size	Non-respondents	Ascertainment of the exposure	The subjects in different outcome groups are comparable	Assessment of the outcome	Statistical test	
Macias et al. (49)	*	*	*	*	**	*	*	8
Lopez-Escamez et al. (19)	*	*	*	*	**	*	*	8
Korres e t al (18).	*	*	*	*	**	*	*	8
Moon et al. (55)	*	*	*	*	**	*	*	8
Pollak et al. (50)	*	*	*	*	*	*	*	7
Korres et al. (56)	*	*	*	*	**	*	*	8
Wee (44)	*	*		*	*	*	*	6
Ahn et al. (57)	*	*	*	*	*	*	*	7
Do et al. (58)	*	*	*	*	**	*	*	8
Lee et al. (59)	*	*	*		*	*	*	7
Balatsouras (20)	*	*		*	*	*	*	6
Soto-Varela et al. (40)	*	*	*	*	*	*	*	7
Shim et al. (41)	*	*	*	*	*		*	6
Silva et al. (60)	*	*	*	*	**	*	*	8
Song et al. (61)	*	*	*	*	**	*	*	8
Brodsky et al. (62)	*	*	*	*	*	*	*	7
Power et al. (21)	*	*	*	*	**	*	*	8
Ouchterlony et al. (63)	*	*	*	*	**	*	*	8
Power et al. (64)	*	*	*	*	**	*	*	8
Si et al. (65)	*	*		*	*	*	*	6
Wang et al. (66)	*	*	*	*	**	*	*	8
Zhang et al. (67)	*	*	*	*	*	*	*	7

The number of ^*^ indicates the score on each sub-component; shaded cell indicates no scores.

### Clinical characteristics of individuals with multiple canal BPPV

A total of 22 articles were included in the present systematic review. These publications documented 5,196 patients diagnosed with BPPV, of which 513 had MC-BPPV, leading to an overall incidence of 9.87%. Out of these 513 individuals, 333 exhibited involvement of multiple canals on the same side, whereas 180 individuals exhibited bilateral involvement (either the same canal on both sides or different canals on either side), constituting an incidence of 64.91% for MC-BPPV and 35.08% for B-BPPV among the multiple canal involvements. In the present systematic review, the combined outcomes of both MC-BPPV and B-BPPV are henceforth referred to as MC-BPPV. Among the studies reporting the aetiologies of MC-BPPV (13 out of 22 articles), most instances of MC-BPPV were identified due to idiopathic causes (68.86%) and head trauma-related precipitations (16.98%). The other less prevalent causes of MC-BPPV included BPPV secondary to viral infections (6.60%), Meniere’s disease (2.83%), migraine (1.88%), vestibular neuritis (0.94%), and otitis media (1.88%) (18, 20, 21, 41, 44, 45, 50, 56–61, 65). The core characteristics of each study were extracted and are depicted in Table 2.

**Table 2:** Core characteristics of the studies included in the systematic review.

Author(s) of the study and Year of publication	Mean age/range (years)	*N*			Tests used for diagnosis		Maneuvers used for treatment	Treatment efficacy							
		SC-BPPV	MC-BPPV		Dix -Hallpik e test	Supine roll test		Success rate in Session I (*N*)		Recurrence rate (*N*)		Unresolved (*N*)		Canal conversion (*N*)	
			U	B											
								SC	MC	SC	MC	SC	MC	SC	MC
Macias et al. (49)		245	13		Y	Y	CRP	189	4						
Lopez-Escamez et al. (19)	28–73	56	7	7	Y	Y	EM & LM	46	10			10	4		
Korres et al. (18)	59.9 ± 12	145	3	10	Y	Y	EM & VM	125	0	14	5	10	2		
Moon et al. (55)	54.8 ± 14	1,608	84		Y	Y	CRP	1,134	57						
Pollak et al. (50)	31–82	30	12	16	Y	Y	EM			10	5				
Korres et al. (56)		188	5	11	Y	Y	EM & VM	165	0	14	2				
Wee (44)	57.l		2	6	Y	Y	EM & BM		7						
Ahn et al. (57)	55.3 ± 15.9	35	3	1	Y	Y	EM & BM								
Do et al. (58)	51.5 ± 16.3	125	13		Y	Y	EM & BM			40	6				
Lee et al. (59)		8	1		Y	Y	CRP								
Balatsouras (20)	M:60.4	11		21	Y	Y	EM, BM, GM, & REM			5		1			
	F:6.8														
Soto-Varela et al. (40)	63	542	10	36	Y	Y	EM & LM	497	42	12	13				
Shim et al. (41)	54.3	1,005	44	5	Y	Y									
Silva et al. (60)	56.7 ± 15.3	100	1		Y	Y	EM, SM, BM, & BE	77	1	11		4			
Song et al. (61)	57 ± 13	210	9	2	Y	Y	EM, SM, & BM	181	1			4	3		
Brodsky et al. (62)	13.4 ± 3.4	138	40	22	Y	Y	EM, SM, GM, YM, BM, & REM	40		20					10
Power et al. (21)	69	79	5	8	Y	Y	EM & BM	35	2		1		1		
Ouchterlony et al. (63)		16		5	Y	Y	EM	5	2			3	1		
Power et al. (64)	61.8 ± 15.2	296	4	14	Y	Y	EM, SM, GM, YM, BM, & BE	228	10						1
Si et al. (65)	64.2 ± 13.9		41		Y	Y	EM, SM, GM,YM, &BM		35				6		
Wang et al. (66)		347	12		Y	Y	EM & BM	280	3						
Zhang et al. (67)	49.1 ± 14.9	12	24	16	Y	Y	EM, GM, & BM	14							5

“SC,” Single canal; “MC,” Multiple canal; “U,” Unilateral;·“B,” Bilateral;·“Y,” Yes; “EM,” Epley ‘s maneuver; “SM,” Semont’s maneuver; “BM,” Barbecue roll maneuver; “LM,” Lempert ‘s maneuver; “GM,” Guffoni maneuver; “VM,” Vannucchi maneuver; “YM,” Yacovino maneuver; “REM,” Reverse Epley’s maneuver; “BE,” Brandt Daroff exercises; Shaded cell indicates information not available.

The results of the Dix-Hallpike test and Supine roll test were used for the diagnosis in all 22 articles. With respect to the management of individuals with MC-BPPV, the first line of treatment initially focused on the canal associated with the worst symptoms and the most intense nystagmus. This was estimated based on the nystagmus characteristics. The treatment for the less symptomatic canal was done after that. To treat various canals, a vast majority of studies relied on a combination of the canalith repositioning maneuvers, especially the modified Epley’s maneuver, alongside other maneuvers.

### Management of individuals with multiple canal BPPV

#### The success rate of CRPs in MC BPPV

Session-by-session information pertaining to the efficacy of treatment for MC-BPPV patients was reported in 14 out of 22 articles (18, 21, 40, 44, 45, 49, 55, 56, 60, 61, 63–66). Of the 295 individuals with MC-BPPV reported in these articles, 174 individuals (58.9%) were effectively treated in one session, whereas 54 (18.3%) individuals and 13 (4.4%) individuals required two and three sessions, respectively for achieving a symptom-free state. There were a few instances (18.4%) of failure of CRP in eliminating symptoms.

#### The recurrence rate after CRPs in MC-BPPV

Four studies reported the recurrence rate of BPPV after successful CRPs in individuals with MC-BPPV (18, 40, 50, 58). In the studies, the recurrence rate typically had a follow-up interval of 15 days to 2 years. Of 97 individuals with MC-BPPV, 29 (29.8%) had a recurrence of symptoms during the above-mentioned follow-up period. Canal conversion was another phenomenon reported in three studies in individuals with MC-BPPV (62, 64, 67). Due to canal conversion, BPPV symptoms persisted in 16 out of 104 individuals (15.38%) after the treatment procedures.

### Efficacy of intervention strategies in multiple canal BPPV

To understand the efficacy of various intervention strategies in individuals with MC-BPPV, the success rates were compared across different intervention strategies. Two studies considered Epley’s maneuver along with the Vannuchi maneuver (EM + VM) (18, 56) and five studies considered Epley’s maneuver along with the Barbecue maneuver/Lempert maneuver (EM + BM) (21, 40, 44, 45, 66) as a treatment option. Of 26 individuals treated with the EM + VM strategy, none turned asymptomatic after the first session of treatment. However, studies that used the EM+ BM strategy had 93 individuals and 64 (68.8%) of them were free from symptoms after the first session of treatment. There was a significant difference in success rate between the EM+ BM and EM+ VM combinations (Z = 6.22, *p* < 0.001, equality of test for proportions) after the first session. Nevertheless, when the efficacy of treatment is compared between these two strategies for the individual to be completely asymptomatic irrespective of the number of sessions, there was no significant difference between the EM + VM and EM + BM strategies (*Z* = 0.32, *p* = 0.74, equality of test for proportions).

### Comparison of treatment efficacy between single canal BPPV and multiple canal BPPV

**Figure 2:**
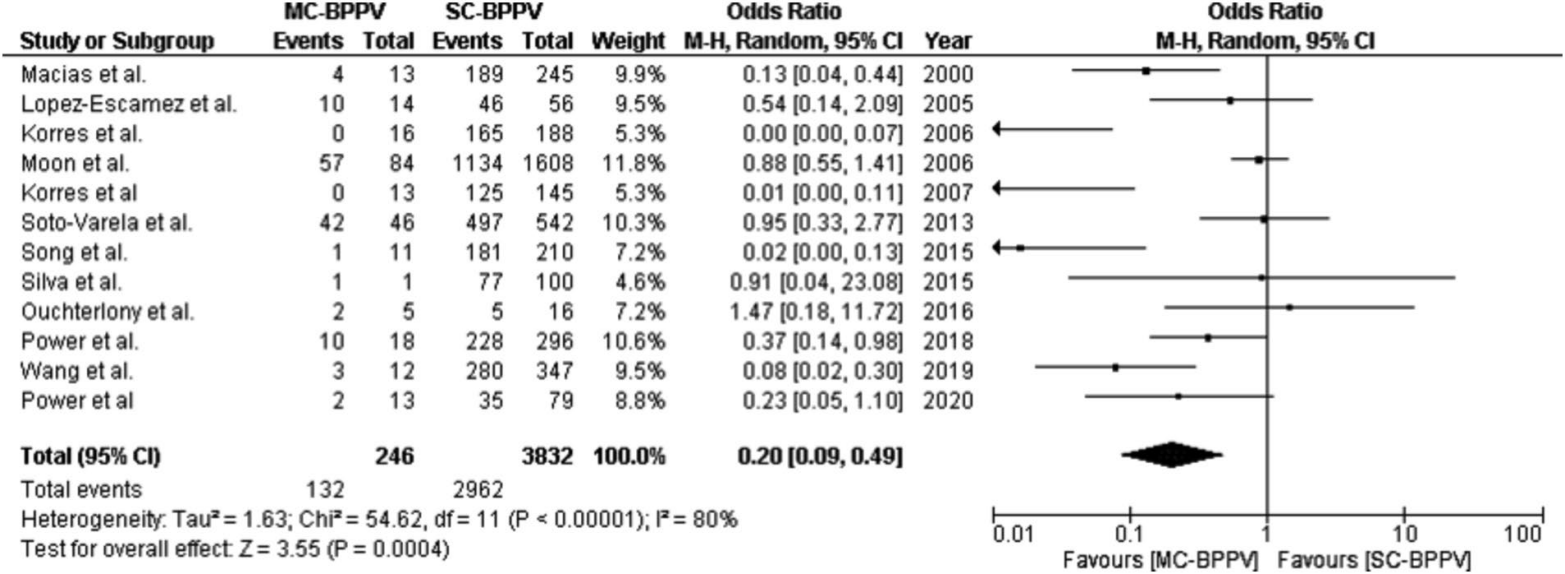
A forest plot for comparison of treatment efficacy in a single session between single-canal BPPV and multiple-canal BPPV.

A total of 12 articles detailed the treatment effectiveness of SC-BPPV and MC-BPPV (18, 21, 40, 45, 49, 55, 56, 60, 61, 63, 64, 66). These studies reported 246 patients with MC-BPPV and 3,832 patients with SC-BPPV. Among those with SC-BPPV, 2,962 (77.29%) individuals reported being symptom-free after one CRP session, whereas 132 (53.65%) individuals with MC-BPPV had no symptoms after the first session. The results of the second session showed an overall resolution of symptoms in 3,521 (91.8%) and 183 (74.4%) individuals with SC-BPPV and MC-BPPV, respectively. At the end of the third session, the symptoms were resolved in 3,743 (97.6%) individuals with SC-BPPV and 206 (83.7%) with MC-BPPV. On the equality of test for proportions, a significantly higher success rate was found for SC-BPPV than MC-BPPV after one session (Z = 8.39, *p <* 0.001), two sessions (Z = 9.21, *p <* 0.001), and three sessions (Z = 12.10, *p <* 0.001). Figure 2 shows a forest plot for comparison of the treatment efficacy of session 1 between individuals with SC-BPPV and MC-BPPV. The success rates were significantly higher in SC-BPPV than in the MC-BPPV (OR = 0.20, 95% CI = 0.09–0.49, *p* = 0.0004).

**Figure 3:**
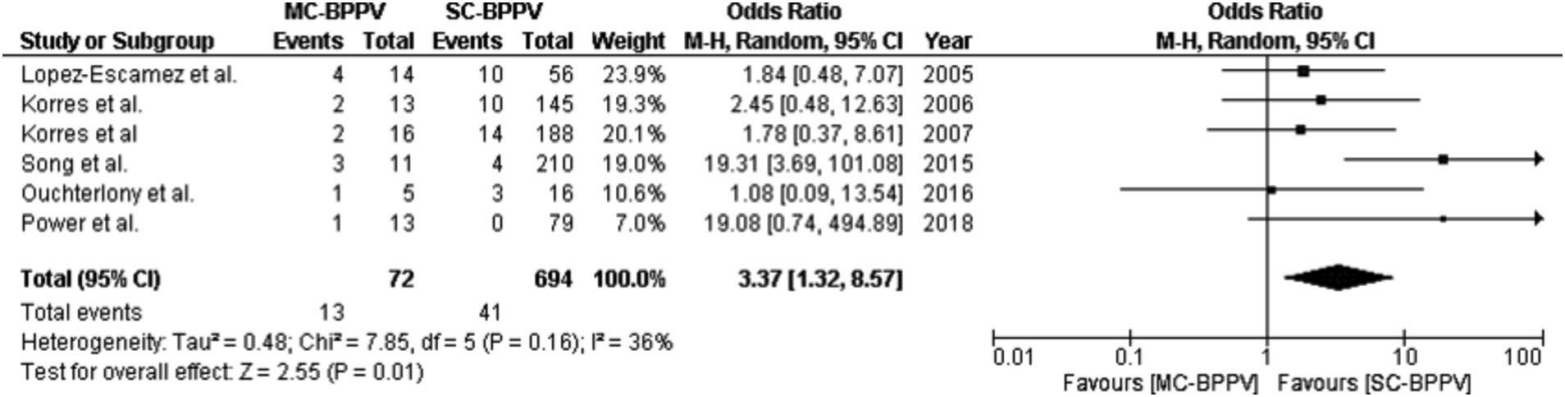
A forest plot for comparison of the number of individuals with unresolved symptoms between single-canal BPPV and multiple-canal BPPV.

In six out of 22 studies, 41 (5.90%) individuals with SC-BPPV and 13 (19.4%) with MC-BPPV did not improve even after multiple CRP treatment sessions (18, 21, 45, 56, 61, 63). On statistical analysis, there was only a marginally significant difference in the proportion of unresolved cases even after multiple CRP sessions between SC-BPPV and MC-BPPV (Z = 3.83, *p <* 0.001, equality of test for proportions). Figure 3 shows a forest plot for comparison of unresolved cases in SC-BPPV and MC-BPPV. The unresolved cases were significantly higher in MC-BPPV than in SC-BPPV (OR = 3.37, 95% CI = 1.32 – 8.57, *p* = 0.01).

In terms of the recurrence rate, four out of 22 studies reported a recurrence rate in 76 out of 842 (9.02%) individuals with SC-BPPV, as against 29 out of 100 (29.0%) individuals with the MC-BPPV (18, 40, 50, 56, 58). On statistical analysis, individuals with SC-BPPV revealed a significantly lesser recurrence rate than the individuals with MC-BPPV after the initial CRP session (Z = 6.00, *p <* 0.001, equality of test for proportions).

## Discussion

Benign paroxysmal positional vertigo is the most common peripheral vestibular disorder accounting for nearly a third of the vestibular disorder. The SC-BPPV is several folds more common than the MC-BPPV. In clinical practice, the MC-BPPV includes either the involvement of two or more canals on the same side or both sides. Among 22 articles included in this systematic review, 513 individuals were diagnosed with MC-BPPV. Out of these, 333 individuals had multiple canal involvement on the same side and 180 individuals had bilateral canal involvement. Thus, the overall incidence of MC-BPPV was 9.87% which falls well within the range of MC-BPPV reported previously (14, 40, 41, 50).

Out of 513 individuals with MC-BPPV, 64.91% were identified as having multiple canal involvement on the same side and 35.08% were bilateral canal involvement. The higher incidence of multiple canal involvement on the same side is in accordance with the previous reports (14, 45, 68, 69). The probable reason for the higher incidence of multiple canal involvement on the same side could be attributed to the etiological factors of MC-BPPV. The etiological factors like Meniere’s disease (70–72) and vestibular neuritis (73), which are commonly associated with MC-BPPV, are more often unilateral and rarely bilateral. Bilateral involvement could be primarily attributed to bilateral damage instigated by traumatic brain injury (74).

### The success rate and recurrence rate of CRPs in MC-BPPV

Canalith repositioning procedures have a high success rate provided that the diagnosis is accurately obtained, and treatment is specific to the canals involved (75). The success rate of CRPs in MC-BPPV was 81.6% after three or more sessions. However, CRPs failed to resolve BPPV symptoms in 18.40% of individuals with MC-BPPV even after three or more sessions. Additionally, 29.8% of individuals with MC-BPPV had a recurrence of symptoms. It has been observed that more maneuvers performed during the first treatment session due to multiple canal involvement could possibly increase the recurrence rate (76). Nevertheless, more than one maneuver is a need that cannot be avoided in the management of individuals with MC-BPPV. In addition, while there is no known explanation for the failure or recurrence rate, another possible factor could be the etiology (77, 78). Specific etiologies such as trauma, labyrinthitis, Meniere’s disease, etc. could make the otolithic macula more susceptible to dislodging of fresh otoconia particles and degenerative changes within them could render the otolith organs less capable of assimilating the returning otoconia debris from the semicircular canals. This would mean that the otoconia particles would remain loose and enter the same or another canal during favorable head positions. A study by Otsuka et al. has shown that if the macula is deprived of the gelatinous mass, the otoconia crystals take longer to assimilate and in about 12–13% of cases, do not assimilate even after 5 min of reaching the otolith organs (79). Since the pathologies associated with MC-BPPV have chances of large-scale erosion of the macula, it could be hypothesized that failure of resolution or recurrence could be higher due to this. However, more evidence needs to be gathered to prove this hypothesis. Some of the other studies have also listed possible reasons such as additional vestibular pathology (50), lesser number of CRP sessions in comparison to the number of canals affected (78), increased likelihood of canal conversion (62, 64, 67), and lesser time gap between the treatment sessions (80).

### Efficacy of intervention strategies in multiple canal BPPV

The comparison between the intervention strategies in individuals with MC-BPPV showed that the EM + BM outperforms the EM + VM after one session. The differences in success rate between these two strategies could be attributed to the procedural differences in achieving different head and body positions between the BM and VM. VM is based on gravity which requires repeating the procedure multiple times to ensure the otoconia particles reach the utricle. In the present systematic review, it has been observed that none of the individuals with MC-BPPV resolved symptoms in the first session using the EM + VM strategy. The results are in accordance with the earlier reports on SC-BPPV where repeated performances of the procedure (usually 5–10 times) are required for a successful treatment using VM (81–83). Whereas, the BM facilitated to clear spontaneously and accelerate the recovery in comparison to the VM and Gufoni maneuver (84). However, both these strategies exhibited similar outcomes on success rate when considered over multiple sessions.

### Comparison of treatment efficacy between single canal BPPV and multiple canal BPPV

On comparison of response to CRPs, it was found that a single session success rate was significantly lower and the failure to resolve after repeated trials significantly higher in MC-BPPV than the SC-BPPV. While this was not true in all studies, the majority resonated with these results even as individual studies. This may imply that the success rate could be dependent on the number of canals involved in the BPPV condition. The possible reasons for the reduced success rate in MC-BPPV than SC-BPPV could be attributed to the complex nature of MC-BPPV. While no study has clearly mentioned the number of individuals with canalolithiasis and cupulolithiasis and the proportion of individuals in whom canalolithiasis was evidenced in one canal and cupulolithiasis in the other canal, this may be a possible factor. The repositioning maneuvers are more effective in individuals with purely canalolithiasis than only cupulolithiasis or mixed types of BPPV (65). Individuals with MC-BPPV can have canalolithiasis and cupulolithiasis type of BPPV (mixed type) which could reduce the success rate or even increase the chances of canal conversion.

The recurrence rate can be defined as the re-occurrence of symptoms after initial successful CRP treatment. It was found to be lesser in individuals with SC-BPPV than the individuals with MC-BPPV. The possible reason for the higher recurrence rate in MC-BPPV could be due to the number of maneuvers and number of sessions required for the treatment of individuals with MC-BPPV (76). Due to the complex nature of the disease, the treatment required is higher when compared to SC-BPPV. Thus, repeatedly undergoing different maneuvers in the same session or different sessions could have disturbed the otoconia crystals and could have resulted in the recurrence of the symptoms.

Age could be another possible reason for the reduced success rate in individuals with MC-BPPV. In elderly individuals, it has been observed that their calcium metabolism is abnormal and otolith particles are larger than young adults. When such large particles enter the semicircular canal, they may be difficult to maneuver for their ouster from the canal due to a narrowed remaining part of the semicircular canal and reduced endolymph fluid velocity which can alter the natural dynamic property of the system. Also, the reduced body flexibility and poor coordination during body movement will reduce the ability to achieve optimum speed and position during the maneuvers which in turn reduces the rate of success (65). Only 1 study has depicted a significant difference in the mean age of individuals in SC-BPPV and MC-BPPV. Individuals with MC-BPPV were older than the individuals with SC-BPPV (40). However, a statistical comparison with age as a factor was not possible with data from a single study in order to prove our hypothesis. Nevertheless, we cannot deny the fact that age is a prognostic factor for successful treatment.

The proportion of unresolved symptoms was 5.90% in the SC-BPPV group, which was significantly lower than that in the MC-BPPV (19.04%). The increase in the number of treatment sessions and a greater number of unresolved cases in MC-BPPV could be due to the etiology of the BPPV (85) and the anatomical changes in different pathologies. For example, in individuals with MC-BPPV secondary to Meniere’s disease, a dilated saccule could result in partial obstruction of the semicircular canal due to the anatomical changes induced by the endolymphatic hydrops. This obstruction can prevent the otoconia particles from returning to the vestibule (86). This could result in a higher proportion of cupulolithiasis secondary to MD and result in a higher frequency of repositioning failures in this group of BPPV. In cases of head trauma, the otolith membrane could be potentially disrupted leading to a predisposition for loose otoconia ready to fall into the SCC during the slightest favorable conditions. This can result in frequent symptom reoccurrences even after a significant number of treatment sessions. Thus, secondary damage to the saccular or utricular macula due to head trauma could be another reason for unresolved cases of MC-BPPV (87). Animal studies have shown that when the macula is intact, the free-floating otoconia debris assimilates into the macula after returning to it. However, when there is partial degeneration of the macula, the otoconia debris takes up to 3 min to get absorbed or settle into the utricle. The situation is worse when the macula has completely degenerated. In such a scenario, the debris takes more time to get absorbed, and there is only an 87.5% chance of complete stabilization (79). Thus, we can hypothesize that the cause of treatment failure in cases of MC-BPPV could be due to degeneration of the utricular macula in those individuals.

During the treatment, there were reports of canal switches in a few individuals with MC-BPPV. Out of 120 MC-BPPV cases, 16 had canal switches reported in three studies (62, 64, 67). This indicates that the treatment of MC-BPPV comes with a heightened chance of a canal switch, i.e., the otoconia moves into another canal instead of going to the vestibule. This results in the occurrence of BPPV symptoms due to stimulation of the healthy canal. The most common canal switch is from a lateral canal to the posterior canal. The reason for the canal switch could be repeated Epley’s maneuver to obtain negative results on the Dix-Hallpike test (64, 88). The other possible reason for canal conversion is the anatomical site of the common crus formed by the joining of the anterior and posterior semicircular canal. In the supine position, the otoconia are more likely to enter the common crus, hence while turning from one side to the other during the treatment of LC-BPPV, the otoliths may fall off spontaneously into the common crus which results in the development of BPPV symptoms in the anterior or posterior canal.

## Conclusion

This study offers insight into the real world of BPPV management in SC-BPPV and MC-BPPV. From this study, it is evident that CRP provides rapid and long-lasting relief of BPPV in most patients. However, in a small subgroup of individuals like the multiple canal BPPV, repeated treatment may be needed, and the risk of recurrences is higher than the SC-BPPV. Thus, the MC-BPPV variant has a significant effect on treatment outcomes due to the longer duration of treatment and a greater number of treatment sessions compared to SC-BPPV.

## Data availability statement

The original contributions presented in the study are included in the article/supplementary material, further inquiries can be directed to the corresponding author.

## Author contributions

MA: Conceptualization, Formal analysis, Investigation, Methodology, Project administration, Resources, Supervision, Validation, Writing – original draft, Writing – review & editing. NS: Conceptualization, Data curation, Formal analysis, Investigation, Methodology, Project administration, Resources, Supervision, Writing – original draft, Writing – review & editing. MAA: Conceptualization, Formal analysis, Investigation, Methodology, Resources, Validation, Visualization, Writing – original draft, Writing – review & editing. NJ: Data curation, Formal analysis, Investigation, Methodology, Resources, Software, Validation, Visualization, Writing – review & editing. RR: Data curation, Formal analysis, Investigation, Methodology, Resources, Software, Validation, Visualization, Writing – review & editing.
